# Should We Use Clinician Self-Report to Tailor Implementation Strategies? Predicting Use of Youth CBT with Clinician Self-Report Versus Direct Observation

**DOI:** 10.1007/s10488-024-01421-y

**Published:** 2024-11-02

**Authors:** Simone H. Schriger, Steven C. Marcus, Emily M. Becker-Haimes, Rinad S. Beidas

**Affiliations:** 1 Department of Psychology, University of Pennsylvania, 425 S. University Avenue, Philadelphia, PA 19104 USA; 2 Department of Psychiatry, Perelman School of Medicine, University of Pennsylvania, Philadelphia, USA; 3 Hall Mercer Community Mental Health, University of Pennsylvania Health System, Philadelphia, USA; 4 Department of Medical Social Sciences, Feinberg School of Medicine, Northwestern University, Chicago, USA

**Keywords:** Self-report, Implementation science, Community mental health, Cognitive behavioral therapy, Youth mental health

## Abstract

Developing tailored implementation strategies to increase the use of evidence-based practice (EBP) requires accurate identification of predictors of their use. However, known difficulties with measuring EBP use complicates interpretation of the extant literature. In this proof-of-concept study, we examined whether the same predictors of use of cognitive behavioral therapy (CBT) are identified when CBT use is measured with clinician self-report compared to direct observation. We examined four candidate predictors of CBT use - clinician participation in an EBP training initiative, years of experience, caseload, and employment status - in a sample of 36 clinicians (64% female; 72% White and 28% Black) from 19 community mental health agencies treating youth in greater Philadelphia. CBT use was captured for 100 unique client sessions (M = 2.8 recorded sessions per clinician) through both clinician self-report and direct observation, using parallel measures. We used three-level (client, clinician, and agency) regression models with random intercepts to estimate the relationship between each predictor variable and CBT use in both measures and compared the magnitude and direction of each model across self-report and direct observation using *z-*tests. There was no alignment for any of the four candidate predictors between predictive relationships identified by self-report compared to those identified by direct observation. The findings in this study extend literature documenting limitations of using clinician self-report to capture clinician behavior and suggest that even the characteristics that *predict* higher self-reported CBT use do not align with (and often are discordant with) those that predict directly observed CBT use. This raises questions about the utility of relying on self-reported use to inform implementation strategy design.

Increasing clinician delivery of evidence-based practice (EBP) is an active process that often relies on the use of implementation strategies (i.e., tailored multilevel supports or techniques used to change behavior; Proctor et al., 2013) to bolster use. Challenges associated with measuring what occurs within a therapy session complicate our ability to accurately identify mutable targets for implementation strategies (Bond & Drake, 2020; Brookman-Frazee et al., 2021; McLeod et al., 2009; Schoenwald, 2011). To date, most studies examining predictors of clinician EBP use have relied on self-report data, rather than direct observation, the latter of which typically involves coding live or recorded sessions. Data derived through observational coding is often considered a proxy for what occurs in session (McLeod et al., 2023). Self-report is a convenient approach to indexing what takes place in session; it is quick to complete, inexpensive, and non-intrusive. Because of this, clinician self-report is a commonly used method to capture session content in routine practice settings and particularly appealing in resource-limited settings such as community mental health (Brookman-Frazee et al., 2021; Hogue et al., 2022). However, self-report has known limitations. While some studies (e.g., Hogue et al., 2015) suggest clinicians can reliably report on their practices, a plethora of studies suggest it may have limited concordance with direct observation and often results in significantly inflated estimates of clinician EBP use (e.g., Becker-Haimes et al., 2022; Brosan et al., 2008; Creed et al., 2016; Hurlburt et al., 2010; Martino et al., 2009; McLeod et al., 2023). For example, in a recently completed randomized controlled trial that tested the accuracy of three alternate measurement strategies (including self-report) compared to direct observation, self-report scores differed significantly from those captured using direct observation, with self-report scores being the most discrepant of the three measurement types tested (Becker-Haimes et al., 2022).

While the challenges in capturing clinician behavior in session have been well characterized in previous work, to our knowledge, no studies to date have assessed whether there are differences in predicting EBP use as a function of measurement type. Given the relative pervasiveness of self-report in research and clinical practice as a tool to capture session content, this study seeks to understand whether predictors of EBP use with youth align when measured by self-report as compared to direct observation. Despite known challenges with concordance between self-report and direct observation at the level of individual sessions, if predictors of EBP use are generally consistent across these two forms of measurement, this may provide increased confidence for using self-report data to understand what drives EBP use, which could inform implementation strategy design. In other words, even if at the level of individual sessions, self-report and direct observation ratings diverge, if both measurement types point to similar *patterns* in EBP use (e.g., clinicians with a lower caseload use more EBP strategies) then self-report data could nonetheless be used on a broader scale to identify fruitful implementation strategy targets. Conversely, if predictors are not the same across measurement types, this suggests that patterns found using self-report data may not align with those found using direct observation, which raises major concerns about the utility of relying on self-report data to inform implementation strategy design. For example, consider a scenario where we found an association between clinician knowledge and EBP use using self-report (e.g., more knowledgeable clinicians report using more EBP) and thus decided to tailor implementation strategies based on the knowledge of the clinician. If this association did not also hold true when measured via direct observation (e.g., there was no significant relationship between knowledge and EBP use, or if the opposite predictive relationship was found), implementation strategies based on knowledge may at best be misdirected and at worst, an unhelpful use of limited resources aimed at improving EBP use.

In the parent trial from which this secondary analysis draws, concordance of self-report and direct observation was found to be poor (whereas scores generated through behavioral rehearsal– simulated role plays– did not differ significantly from those generated through direct observation, and scores captured via chart-stimulated recall - structured interviews with the chart available– did not differ significantly from behavioral rehearsal scores; Becker-Haimes et al., 2022). In this secondary analysis, we examined whether the association between candidate predictors of clinician cognitive behavioral therapy (CBT) use (a core psychosocial EBP; Dorsey et al., 2017; Higa-McMillan et al., 2016; Hofmann et al., 2012; Weisz et al., 2017), and clinician CBT use scores differed when measured via self-report compared to direct observation. We focused specifically on self-report given the relative prevalence of this method in research and routine care compared to chart-stimulated recall and behavioral rehearsal, and the fact that this method was the most discrepant from direct observation. Given poor concordance with direct observation scores, we sought to examine whether self-report data could be “salvaged” by using it for the purpose of identifying broader predictive trends. Further, we focused specifically on the self-report arm of the trial in order to minimize the relative number of comparisons.

We approached this study as a proof-of-concept in examining alignment of predictors across multiple measurement types and were selective in choosing candidate predictors in order to minimize multiple comparisons and the likelihood of Type 1 errors. We selected four candidate predictors from the set of variables collected in the parent trial: past participation in one of five system sponsored EBP training initiatives; years of experience; client caseload; and employment status (salaried versus independent contractor). When selecting these variables, we were limited by what was measured in the parent trial, and also considered their salience within the literature and the variability of our data. Within the literature, system-sponsored EBP training, greater years of experience, and lower caseloads have all been associated with greater self-reported CBT use in multiple studies (Becker-Haimes et al., 2017; Beidas et al., 2015, 2019; Frank et al., 2020). Salaried employees also have endorsed more positive attitudes and greater knowledge of CBT compared to independent contractors (Beidas et al., 2016). We expected that despite data showing limited convergence between self-report and direct observation for measuring CBT use, there would be alignment of predictive relationships across both measurement types (i.e., comparable directionality of identified effects).

## Method

This study was approved by the University of Pennsylvania (Approval #834079) and City of Philadelphia (Approval #2016-24) Institutional Review Boards, and all guardians, clients, and clinicians involved in the study provided consent or assent.

### Setting and Procedure

This study leverages data from the randomized controlled trial described above that compared accuracy of three measurement strategies to capture use of youth CBT compared to direct observation (see Becker-Haimes et al., 2022 for details about parent trial). Briefly, clinicians were recruited from 27 community mental health agencies and randomized 1:1:1 to one of three measurement conditions: self-report, chart-stimulated recall, and behavioral rehearsal. All participating agencies were in the Philadelphia tristate area and most agencies had participated in a system-sponsored EBP training initiative to implement one of five CBT-based EBPs for youth (including cognitive therapy, trauma-focused CBT, prolonged exposure, dialectical behavioral therapy, and parent-child interaction therapy; Beidas et al., 2013, 2019; Powell et al., 2016). Data collection occurred between October 2016 and May 2020. Each clinician recorded up to three client sessions, each with a separate client from their caseload. Each session was audio-recorded, stored in a HIPAA compliant platform, and subsequently coded for use of 12 CBT techniques by trained independent raters. Each clinician also completed a second measure aimed to capture CBT use (either self-report, chart-stimulated recall, or behavioral rehearsal) for each recorded session. Clinicians and clients were compensated for their participation. In this secondary proof-of-concept analysis, we draw from the self-report arm of the study to address study aims.

### Participants

Participants were 36 clinicians from the self-report condition of the parent trial (of the 41 clinicians originally randomized to self-report, five were excluded due to: withdrawal (*n* = 1), interruption due to COVID-19 (*n* = 3), and inability to enroll clients (*n* = 1)). These clinicians came from 19 of the 27 sites, and each clinician enrolled a mean of 2.78 recorded sessions included in the study (*SD* = 0.54), for a total of 100 sessions (each with a unique client) across the 36 clinicians (see Becker-Haimes et al., 2022 for CONSORT diagram and details of enrolled participants).

Clinicians were a median of 34 years old (range = 25–78), predominantly identified as female (64%), and all held master’s degrees. Clinicians had a median of 10 years of experience (range = 1–60) and held a median caseload of 20 clients (range = 5–65). Twenty-five clinicians (69%) had participated in a system sponsored EBP training initiative. Just over half of clinicians indicated they were salaried (54%); 46% were independent contractors (and one clinician did not respond). Clinicians identified with a variety of racial backgrounds, with the majority identifying as White (72%; See Table 1).

**Table 1:** Clinician characteristics

Variable *(n* = *36)*	Statistic
Age, median (range) *(n* = *35)*	34 (25–78)
Gender, *n* (%)	
Female	23 (63.9)
Male	12 (33.3)
Prefer not to disclose	1 (2.8)
Hispanic/Latinx ethnicity, *n* (%) *(n* = *34)*	0 (0)
Racial identity^a^, *n* (%) *(n* = *32)*	
Asian or Pacific Islander	0 (0)
Black or African American	9 (28.1)
Other	1 (3.1)
White	23 (71.9)
Master’s-level, *n* (%)	36 (100)
Number of client sessions recorded in study, *M (SD)* Candidate predictor Participated in EBP training initiative^b^, *n(%)* Years of experience, *Median (IQR)* Caseload, *Median (IQR)* Independent contractor^bcd^, *n(%)*	2.78 (0.54) 25 (69.4%) 10.0 (4.0–15.0) 20.0 (12.1–32.8) 16 (45.7%)

^a^Numbers do not add to 100% as clinicians could self-identify with more than one race; ^b^Percentage is listed for binary variables, as they were coded as 0 or 1; ^c^*n*=35 as one clinician did not provide a response; ^d^Those who were not independent contractors were all salaried employees

### Predictor Variables

Four candidate predictor variables - past participation in one of five system-sponsored EBP training initiatives; years of experience; client caseload; and employment status (salaried versus independent contractor) - were captured in the parent trial through a questionnaire that asked clinicians about their demographics and other work-related characteristics, administered at trial enrollment. We selected only four candidate predictor variables to limit the total number of comparisons.

### Measures

#### Direct Observation

To capture CBT use using direct observation, audio recordings were coded using a validated observational coding system, the Therapy Process Observational Coding System Revised Strategies (TPOCS-RS) scale (McLeod et al., 2015). Sessions were coded by one of 11 raters who were trained extensively prior to beginning coding and met regularly to prevent drift (see Becker-Haimes et al., 2022 for details). This measure has shown good internal consistency and validity in multiple studies (McLeod et al., 2015; Smith et al., 2017). Coders rated use of 12 youth CBT techniques using a 7-point Likert scale ranging from 1 (not at all) to 7 (extensively). Interrater agreement on the TPOCS-RS was high on all techniques. The TPOCS-RS yields three aggregate CBT use scores (CBT “maximum,” “mean,” and “count” scores), each of which was designed to capture a slightly different aspect of CBT use (Smith et al., 2017). The collective use of these three metrics captures depth and breadth of CBT delivery, all of which are meaningful for indexing use of CBT (Garland et al., 2006). The CBT maximum score, conceptualized as the “depth” of CBT use, was defined as the single highest coded technique in each session (range = 1–7). The CBT mean score, conceptualized as a combination of depth and breadth, was defined as the mean score of all endorsed (i.e., scored > 1) techniques (range = 1–7). The CBT count score, conceptualized as the “breadth” of CBT use, was defined as the total number of CBT techniques endorsed as present (i.e., rated > 1) in a given session (range = 0–12). It is important to note that lower scores do not always indicate poorer CBT delivery. For example, a clinician who focused extensively on a single CBT technique may have a high “CBT maximum” score due to extensive depth, but a low “CBT count” score due to limited breadth.

#### Self-Report

Clinicians’ self-report ratings were collected using the Therapy Process Observational Coding System Self-Reported Therapist Intervention Fidelity for Youth (TPOCS-SeRTIFY; Becker-Haimes et al., 2021). Designed to parallel the TPOCS-RS to facilitate analysis of concordance with direct observation, this measure asks clinicians to report their use of the same 12 CBT techniques (each of which is operationally defined) on an identical Likert scale ranging from 1 (not at all) to 7 (extensively). Prior to completing the measure, clinicians were given a 30-minute training on its use and were provided with a rating manual containing sample vignettes of clinician behaviors and their associated ratings. Clinicians were asked to complete the self-report measure within 48 h of the client session. Based on initial psychometric analyses, this measure has shown preliminary construct validity, strong item performance, and strong concordance with another established self-report measure (Becker-Haimes et al., 2021). However, it showed poor concordance with direct observation in the original trial (Becker-Haimes et al., 2022), consistent with the larger literature documenting low concordance between self-report and direct observation (e.g., Brosan et al., 2008; Creed et al., 2016; Hurlburt et al., 2010; Martino et al., 2009).

### Statistical Analysis

Rates of missing data in this subsample were extremely low (< 0.01%). To account for the nested nature of the data, three-level (client, clinician, and agency) regression models with random intercepts were conducted using PROC MIXED in SAS Version 9.4 (Singer, 1998). Twelve models estimated the relationship between each of the four candidate predictors (i.e., participation in an EBP training initiative, years of experience, caseload, employment status) and each of the three ways to calculate CBT use (i.e., CBT maximum, mean, and count) first for self-report and then for direct observation. Random intercepts accounted for nesting of clients within clinicians and clinicians within agencies. Primary outcomes were the coefficients and significance tests of the candidate predictor variables in the regression models. A significant *p* value indicated a non-zero relationship between the predictor variable and CBT use score. Secondary follow-up analyses used *z*-tests to determine whether the magnitude and direction of the association between the predictor variable and the CBT use scores significantly differed based on whether CBT use was measured using self-report or direct observation. Alpha was set to 0.05 for all analyses; given the exploratory nature of the analyses, we did not adjust for multiple comparisons (Rothman, 1990).

## Results

Table 2 shows median CBT maximum, mean, and count scores based on self-report and direct observation. Consistent with the primary trial outcomes, self-report scores were higher than direct observation scores across all three CBT use scores, meaning that clinicians self-reported higher use of CBT than was directly observed.

**Table 2:** Descriptive statistics of candidate predictors and CBT use scores

	Median (IQR)	
CBT Use Score	SR (*n* = 100)	DO (*n* = 100)
CBT Maximum Score ^a^	5.0 (5.0–6.0)	3.5 (2.0–4.0)
CBT Mean Score, ^a^	3.86 (3.34–4.14)	2.63 (2.0–3.0)
CBT Count Score ^b^	7.0 (5.0–9.0)	3.0 (2.0–4.0)

*Abbreviations* SR, Self-Report (measured by the TPOCS-SeRTIFY); DO, Direct Observation (measured by the TPOCS-RS) ^a^Possible range = 1–7; ^b^Possible range = 0–12

**Fig. 1:**
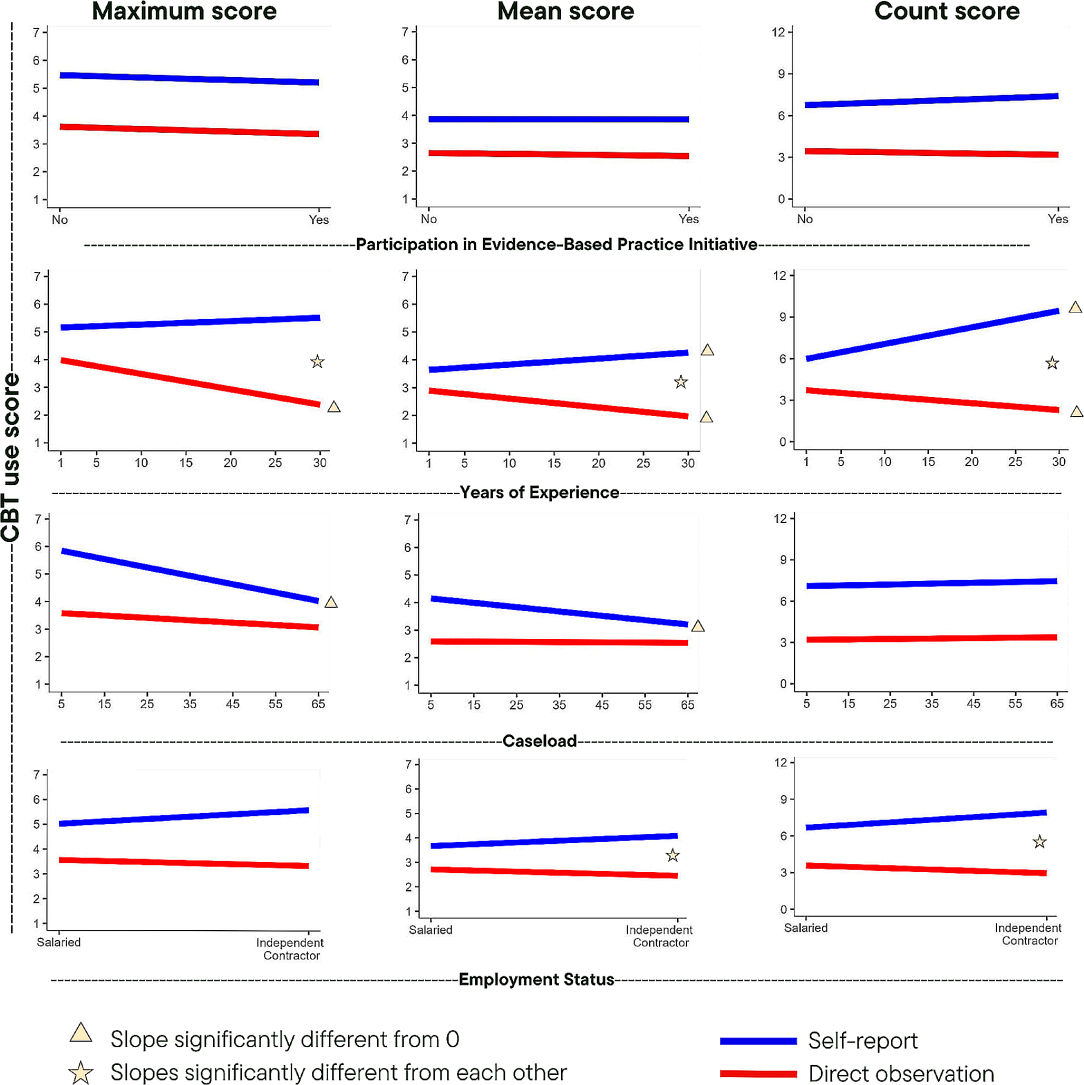
Visualization of Relationships Between Candidate Predictors and CBT Use Each of the 12 panels depicts the relationship between candidate predictor and CBT use score by measurement type, as derived from three-level (client, clinician, agency) regression models with random intercepts. Exact values can be found in Table 3

Table 3 shows results of the 12 predictive models, each of which are depicted in Fig. 1, which illustrates the association between each predictor variable and each CBT score metric for self-report and direct observation.

**Table 3:** Relationships between candidate predictors and CBT use scores ^a^

	SR (*n* = 100)		DO (*n* = 100)		SR vs. DO
	b (SE)	*p*	b (SE)	*p*	*p* ^b^
*Max CBT Score*					
Participated in initiative^c^	-0.27 (0.33)	0.42	-0.27 (0.43)	0.54	> 0.99
Years of experience	0.01 (0.02)	0.43	-0.06 (0.02)	< 0.01*	< 0.01
Caseload	-0.03 (0.01)	< 0.01*	-0.01 (0.02)	0.57	0.23
Independent contractor^d, e^	0.56 (0.31)	0.08	-0.23 (0.44)	0.61	0.14
*Mean CBT Score*					
Participated in initiative^c^	<-0.01 (0.23)	0.98	-0.11 (0.24)	0.66	0.76
Years of experience	0.2 (0.01)	0.04*	-0.03 (0.01)	< 0.01*	< 0.01
Caseload	-0.02 (0.01)	0.04*	<-0.01 (0.01)	0.91	0.18
Independent contractor^d, e^	0.42 (0.21)	0.06	-0.26 (0.24)	0.27	0.03
*Count CBT Score*					
Participated in initiative^c^	0.65 (0.84)	0.44	-0.27 (0.41)	0.51	0.32
Years of experience	0.12 (0.03)	< 0.01*	-0.05 (0.02)	0.01*	< 0.01
Caseload	0.01 (0.03)	0.84	< 0.01 (0.02)	0.85	0.92
Independent contractor^d, e^	1.23 (0.78)	0.12	-0.63 (0.45)	0.17	0.04

*Abbreviations* SR, Self-Report (measured by the TPOCS-SeRTIFY); DO, Direct Observation (measured by the TPOCS-RS); ^a^Coefficients are derived from three-level (client, clinician, agency) regression models with random intercepts using each CBT score as a dependent variable and each candidate predictor as an independent variable; models were run separately for SR and DO scores; ^b^Two-tailed *p* value by *z*-test comparing self-report coefficient from DO coefficient; *p < 0.05, indicating a statistically significant relationship between predictor and CBT use score; ^c^This variable was coded as yes = 1 and no = 0; ^d^This variable was coded as independent contractor = 1 and salaried = 0; ^e^*n*=98 as one clinician (with two client sessions included in analyses) did not provide a response

### Participation in EBP Training Initiative

Contrary to hypotheses, when examined in relation to both self-report and direct observation, clinician participation in a formal, system sponsored EBP training initiative did not relate to CBT use using any of the three use scores (all *p*s > 0.05).

### Clinician Years of Experience

Consistent with prior studies when using self-report, more years of experience predicted more CBT use (Mean CBT Score *b* = 0.2, *SE* = 0.01, *p* = 0.04) and a greater number of CBT techniques used (CBT Count *b* = 0.12, *SE* = 0.03, *p* = < 0.01); however, years of experience did not predict the depth of CBT delivery (CBT Max score *b* = 0.01, *SE =* 0.02, *p* = 0.43*)*. In contrast, when using direct observation, clinicians with more years of experience had lower average CBT use (Mean CBT Score *b* = -0.03, *SE* = 0.01, *p* = < 0.01), lower total count of techniques observed (CBT Count *b* = -0.05, *SE* = 0.02, *p* = 0.01), and lower depth of CBT delivery (CBT Max score *b* = -0.06, *SE* = 0.02, *p* = < 0.01), than those with fewer years of experience. Post-hoc *z*-tests indicated that coefficients for clinician years of experience in models of the maximum, mean, and count CBT scores differed significantly depending on whether they were measured by self-report or direct observation (all *p*s < 0.01).

### Caseload

When caseload was examined in relation to self-reported CBT use, consistent with prior work, higher caseload predicted lower depth of CBT delivery (CBT Max score *b* = -0.03, *SE* = 0.01, *p* = < 0.01) and lower average CBT use (Mean CBT Score *b* = -0.02, *SE =* 0.01, *p* = 0.04); however, client caseload did not predict the number of CBT techniques used (CBT Count *b* = 0.01, *SE* = 0.03, *p* = 0.84). In contrast, when examined in relation to direct observation scores, there was no relationship between caseload and CBT use for any of the three CBT scores (all *p*s > 0.05). No differences were found in post-hoc *z*-tests comparing coefficients for caseload when using self-report versus direct observation for CBT maximum, mean, or count scores (all *p*s > 0.05).

### Employment Status

Contrary to prior work, there were no significant relationships between employment status and CBT use for any of the three score types when measured using self-report or direct observation (all *p*s > 0.05). However, post-hoc *z*-tests indicated that coefficients for employment status in models of both mean and count CBT scores differed significantly when using self-report (which showed a weak, non-significant positive relationship between being an independent contractor and CBT use) and direct observation (which showed a weak, non-significant negative relationship between being an independent contractor and CBT use; *p*s < 0.05).

## Discussion

Accurately identifying predictors of EBP use can be critically useful in designing implementation strategies aimed at increasing clinician delivery of EBP in routine care settings. In this study, we examined alignment between self-reported and directly observed CBT use measurement as it related to the relationships between four candidate predictors and CBT use. Despite documented inaccuracies of clinician self-report as a method of measuring what occurs in session (i.e., clinicians often overestimate use of techniques compared to direct observation), this less expensive and more feasible form of measurement is convenient and will likely continue to be used in research and routine practice. If predictors of CBT use aligned across self-report and direct observation, self-report data could still confidently inform implementation strategy design to increase use of CBT. Unfortunately, we found that across all four candidate predictors, there was no alignment when using self-report and direct observation regardless of how CBT use was measured (i.e., maximum, mean, or count). This has important implications in the context of a field that has often relied upon self-report data when measuring use of EBP in order to design implementation strategies. These data extend existing findings documenting limitations of self-report, suggesting that even the characteristics that *predict* higher self-reported CBT use are different from those that predict higher CBT use when measured by direct observation.

Across our predictive models, we found three types of discordance, all of which have implications for implementation strategy design. First, there were instances in which a variable was predictive of high self-reported CBT use but not predictive of high CBT use when measured using direct observation, such as with client caseload. This pattern has implications for potential misalignment of investment in implementation strategies. For example, if policymakers were to leverage this data and cap clinician caseloads with the intention of improving CBT use, they may not find that this invested effort translates into a measurable change. While such an investment may still have positive benefits (e.g., reducing clinician burnout), it may not translate to improved clinical outcomes. A second pattern of discordance occurred in instances where a variable was not predictive of higher self-report scores but was significantly predictive of higher directly observed CBT use, as occurred with clinician years of experience when use was captured using the CBT maximum score. This pattern is a missed opportunity -- policy makers relying on self-report data might be led to believe that there is no relationship between clinician years of experience and use of CBT, when direct observation suggests that clinicians with greater years of experience actually have lower CBT use compared to those with fewer years of experience. When using the CBT mean and count scores with this same predictor variable, we found a third pattern of discordance in which clinician years of experience significantly predicted both self-report and direct observation scores, but in opposite directions. This pattern could lead policymakers to design implementation strategies that are counterproductive, in that they select the wrong subset of clinicians to target (e.g., targeting clinicians with fewer years of experience when in fact those with greater years of experience are most in need of additional support). Critically, this latter form of discordance had a robust effect; post-hoc *z*-tests indicated that for both the mean and count CBT scores, self-report and direct observation coefficients were significantly different from each other.

Taken together, these findings suggest that using self-report data to inform the design of implementation strategies aimed at increasing EBP use may be ill-advised. At best, such an approach may result in poor investment of resources and/or missed opportunities (i.e., the first and second patterns of discordance we observed) and at worst may risk moving the field farther away from the goal of increasing EBP, as illustrated by the third pattern of discordance. These findings may help explain why we have inconsistencies across the literature in predictors of EBP use, as some studies use self-report while others use direct observation. Further, these results raise questions about relying on conclusions drawn from studies that examining predictors of CBT use using self-report data. When examining predictive relationships based on directly observed CBT use, of the candidate predictors tested in this study, only clinician years of experience was found to be significantly predictive of CBT use. In our sample, clinician years of experience was negatively associated with observed clinician CBT use across CBT maximum, mean, and count scores. This suggests that clinicians with greater years of experience used less CBT than those with fewer years of experience, which contrasts with previous studies relying on self-report data that have found the opposite relationship (e.g., Beidas et al., 2015). Though direct observation scores are an approximation of what occurs in session (and not inherently free from error, or the complete “truth” of what occurs in a session), the extent to which these findings diverge underlines the need for caution when interpreting predictive relationships based on self-report data.

Results should be interpreted within the context of study limitations. First, all clinicians came from the same public behavioral health system and completed the same self-report measure, which may limit generalizability to other systems and measures. Second, the level of CBT use in our sample was low; predictors of self-reported CBT use may be more aligned with observed use in samples where CBT delivery is higher. Third, although across our two measurement conditions we had 200 observations, our sample size of 36 clinicians was relatively small and should be expanded upon in future work in order to bolster statistical power. Fourth, our selection of candidate predictor variables was limited by the data collected within the parent trial, and we were thus unable to include other potential candidate predictor variables that have been identified in literature using self-reported CBT use (e.g., clinician knowledge, attitudes, licensure status, age, self-efficacy) (Becker-Haimes et al., 2017; Beidas et al., 2015; Harned et al., 2013; Okamura et al., 2019; Reid et al., 2018; Wolk et al., 2016) and/or observed CBT use (e.g., clinician theoretical orientation; Brookman-Frazee et al., 2010; Wolk et al., 2016). Finally, this study focuses on a single EBP, CBT, as an exemplar for EBPs more broadly, given its strong empirical support and widespread implementation across community settings. Previous research involving a different EBP that examined concordance between scores generated via self-report compared to direct observation found greater success (Hogue et al., 2015), and thus there may be promise in testing alignment of predictors for other interventions beyond CBT. Further, previous work involving another EBP found that reliability and accuracy of clinician self-report improved following an online training, and it may be that such techniques can be applied to CBT (Hogue et al., 2023). Our study also focused on use of 12 general CBT strategies rather than adherence to a particular protocol. Further, although over two-thirds of clinicians had participated in a system-sponsored EBP initiative, trainings spanned five CBT-based interventions and thus not all clinicians were trained in the same modality. In future research, it may be useful to examine whether there is greater alignment across self-report and direct observation when examining predictors of adherence to a specific CBT protocol for which all clinicians received training.

This study also has notable strengths. To our knowledge, it is the first to assess differences in predictors of EBP use as a function of measurement type. Our findings of discordance between self-report and direct observation caution against relying on self-report data when designing implementation strategies and may advance the field in moving away from measuring clinician CBT use with self-report. Unlike research that has examined specific patterns of disagreement across methods in other areas and found value in a multi-informant approach (e.g., assessing severity of youth psychopathology; De Los Reyes et al., 2015), our findings bolster existing work that has failed to find evidence to support a multi-informant approach to measuring CBT use (McLeod et al., 2023). Additionally, our study has strong ecological validity in that our data come from clinicians embedded within community mental health clinics in a system that has prioritized EBP implementation. Further, this study uses self-report and direct observation measures of the same client session using measures that were designed to parallel one another, adding to the rigor of our findings of discordance. Our findings contribute to the literature on challenges associated with self-report; we found that beyond simply overestimating direct observation scores, self-report scores also have different relationships with predictor variables than do direct observation scores, with no evidence of alignment across any predictors tested. At best, clinician self-reported CBT use may be more accurately conceptualized as a measure of clinicians’ *perception* of their CBT delivery, or perhaps of what they perceive they *should* be reporting (e.g., in a health system that values use of EBP, clinicians with more years of experience may be more inclined to *report* greater CBT use).

Our results point to several additional questions that warrant further research. First, while we focus specifically on the self-report arm of the parent trial, future research should examine whether concordance of predictors is greater when using other methods of indexing CBT use (i.e., chart-stimulated recall and behavioral rehearsal). Second, given the dynamic and bidirectional relationship between predictors at multiple levels of analysis, there is a need for more nuanced methods of examining predictive relationships, including testing for interactions between multilevel predictors and examining potential moderation effects.

Findings from this proof-of-concept study suggest that predictors of youth CBT use are heavily dependent on the type of indexing measures we select. If we want to identify clinician factors that predict greater use of CBT, we should be cautious when relying on self-report data. Our results have robust implications regarding implementation strategy design, particularly in a field that often relies on self-report data; they caution us against designing our implementation strategies based on predictors of high self-reported clinician use of EBP as doing so may not lead to desired changes.

## Data Availability

The data that support the findings of this study are available from the first author upon reasonable request.

## Declarations

### Reporting

We followed the CONSORT reporting guidelines for clinical trials.
