# Correction To: Should We Use Clinician Self-Report To Tailor Implementation Strategies? Predicting Use of Youth CBT With Clinician Self-Report Versus Direct Observation

**DOI:** 10.1007/s10488-025-01434-1

**Published:** 2025-03-01

**Authors:** Simone H. Schriger, Steven C. Marcus, Emily M. Becker-Haimes, Rinad S. Beidas

**Affiliations:** 1 Department of Psychology, University of Pennsylvania, 425 S. University Avenue, Philadelphia, PA 19104 USA; 2 Department of Psychiatry, Perelman School of Medicine, University of Pennsylvania, Philadelphia, USA; 3 Hall Mercer Community Mental Health, University of Pennsylvania Health System, Philadelphia, USA; 4 Department of Medical Social Sciences, Feinberg School of Medicine, Northwestern University, Chicago, USA

**Correction to: Administration and Policy in Mental Health and Mental Health Services Research**

10.1007/s10488-024-01421-y.

In this article, the unmasked citation of “REDACTED” information has been inadvertently missed to update during the article publication. The REDACTED information has been corrected with the updated citation.

The original article has been corrected.

1. [REDACTED] in the sentence beginning “For example, in a recently completed………………” should have read as given below.

## Incorrect Sentence

For example, in a recently completed randomized controlled trial that tested the accuracy of three alternate measurement strategies (including self-report) compared to direct observation, self-report scores differed significantly from those captured using direct observation, with self-report scores being the most discrepant of the three measurement types tested **([REDACTED])**

## Corrected Sentence

For example, in a recently completed randomized controlled trial that tested the accuracy of three alternate measurement strategies (including self-report) compared to direct observation, self-report scores differed significantly from those captured using direct observation, with self-report scores being the most discrepant of the three measurement types tested (**Becker-Haimes et al.**,** 2022**).

2. [REDACTED] in the sentence beginning “In the parent trial from which this secondary……………” should have read as given below.

## Incorrect Sentence

In the parent trial from which this secondary analysis draws, concordance of self-report and direct observation was found to be poor (whereas scores generated through behavioral rehearsal– simulated role plays– did not differ significantly from those generated through direct observation, and scores captured via chart-stimulated recall - structured interviews with the chart available– did not differ significantly from behavioral rehearsal scores; [**REDACTED**]).

## Corrected Sentence

In the parent trial from which this secondary analysis draws, concordance of self-report and direct observation was found to be poor (whereas scores generated through behavioral rehearsal– simulated role plays– did not differ significantly from those generated through direct observation, and scores captured via chart-stimulated recall - structured interviews with the chart available– did not differ significantly from behavioral rehearsal scores; **Becker-Haimes et al.**,** 2022**).

3. In **“Method”** section, [REDACTED] should have read as given below.

## Incorrect Sentence

This study was approved by the **[REDACTED]** (Approval #834079) and **[REDACTED]** (Approval #2016-24) Institutional Review Boards, and all guardians, clients, and clinicians involved in the study provided consent or assent.

## Corrected Sentence

This study was approved by the **University of Pennsylvania** (Approval #834079) and **City of Philadelphia** (Approval #2016-24) Institutional Review Boards, and all guardians, clients, and clinicians involved in the study provided consent or assent.

4. In the subheading “**Setting and Procedure**, [REDACTED] information in two sentences in the first paragraph should have read as given below.

## Incorrect Sentence

This study leverages data from the randomized controlled trial described above that compared accuracy of three measurement strategies to capture use of youth CBT compared to direct observation (see **[REDACTED]** for details about parent trial).

## Corrected Sentence

This study leverages data from the randomized controlled trial described above that compared accuracy of three measurement strategies to capture use of youth CBT compared to direct observation (see **Becker-Haimes et al.**,** 2022** for details about parent trial).

## Incorrect Sentence

All participating agencies were in the **[REDACTED]** and most agencies had participated in a system-sponsored EBP training initiative to implement one of five CBT-based EBPs for youth (including cognitive therapy, trauma-focused CBT, prolonged exposure, dialectical behavioral therapy, and parent-child interaction therapy; **[REDACTED]**).

## Corrected Sentence

All participating agencies were in the **Philadelphia tristate area** and most agencies had participated in a system-sponsored EBP training initiative to implement one of five CBT-based EBPs for youth (including cognitive therapy, trauma-focused CBT, prolonged exposure, dialectical behavioral therapy, and parent-child interaction therapy; **Beidas et al.**,** 2013**,** 2019; Powell et al.**,** 2016)**.

5. The second sentence “These clinicians came from 19 of the 27 sites…………….”in the first paragraph under the subheading **“Participants”** should have read as given below.

## Incorrect Sentence

These clinicians came from 19 of the 27 sites, and each clinician enrolled a mean of 2.78 recorded sessions included in the study (*SD* = 0.54), for a total of 100 sessions (each with a unique client) across the 36 clinicians (see **[REDACTED]** for CONSORT diagram and details of enrolled participants).

## Corrected Sentence

These clinicians came from 19 of the 27 sites, and each clinician enrolled a mean of 2.78 recorded sessions included in the study (*SD* = 0.54), for a total of 100 sessions (each with a unique client) across the 36 clinicians (see **Becker-Haimes et al.**,** 2022** for CONSORT diagram and details of enrolled participants).

6. In “Measures” section, under the sub heading “Direct Observation”, the second sentence in the first paragraph should have read as given below.

## Incorrect Sentence

Sessions were coded by one of 11 raters who were trained extensively prior to beginning coding and met regularly to prevent drift (see **[REDACTED]** for details).

## Corrected Sentence

Sessions were coded by one of 11 raters who were trained extensively prior to beginning coding and met regularly to prevent drift (see **Becker-Haimes et al.**,** 2022** for details).

7. In “**Measures**”section, under the sub heading “**Self-Report**”, the last sentence in the last paragraph should have read as given below.

## Incorrect Sentence

However, it showed poor concordance with direct observation in the original trial **([REDACTED])**, consistent with the larger literature documenting low concordance between self-report and direct observation (e.g., Brosan et al., 2010; Martino et al., 2009).

## Corrected Sentence

However, it showed poor concordance with direct observation in the original trial (**Becker-Haimes et al.**,** 2022**), consistent with the larger literature documenting low concordance between self-report and direct observation (e.g., Brosan et al., 2010; Martino et al., 2009).

8. In “**Declarations**” section, under the “**Ethical Approval**” subheading, the [REDACTED] information should have read as given below.

## Incorrect Statement

Ethics approval was granted by the **[REDACTED]** (Approval #834079) and **[REDACTED]** (Approval #2016-24) Institutional Review Boards, and all guardians, clients, and clinicians involved in the study provided consent or assent.

## Corrected Statement

Ethics approval was granted by the **University of Pennsylvania** (Approval #834079) and **City of Philadelphia** (Approval #2016-24) Institutional Review Boards, and all guardians, clients, and clinicians involved in the study provided consent or assent.

